# Antibiotic-resistant bacteria and resistance-genes in drinking water source in north Shoa zone, Amhara region, Ethiopia

**DOI:** 10.3389/fpubh.2024.1422137

**Published:** 2024-09-06

**Authors:** Tsegahun Asfaw Abebe, Demissew Shenkute Gebreyes, Bizuneh Asfaw Abebe, Berhanu Yitayew

**Affiliations:** ^1^Department of Medical Laboratory Science, College of Health Science, Debre Berhan University, Debre Berhan, Ethiopia; ^2^School of Civil Engineering, Ethiopian Institute of Technology-Mekelle, Mekelle University, Mekelle, Ethiopia

**Keywords:** bacteria, antibiotic-resistance, resistance-gene, drinking water source, Ethiopia

## Abstract

**Background:**

The growing number of antimicrobial-resistant bacteria in a range of environments poses a serious challenge to infectious disease prevention. Good water quality is critical to human health and has a direct impact on a country’s socio-economic growth. Therefore, assessing the bacteriological quality of drinking water provides benchmark data and provides insight into the development of further protection and treatment measures.

**Methods:**

A cross-sectional study was conducted from February 1, 2022, to September 31, 2023, in the diarrhea hotspot areas of North Shewa Zone (Minjar-Shenkora and Mojana-Wedera districts). Water samples were collected from drinking water sources (hand-pumps, boreholes, wells, spring water and ponds) to assess the quality following WHO guidelines. The collected water samples were processed for bacterial isolation, antimicrobial susceptibility testing, and detection of antimicrobial resistance genes. Data were entered and analyzed using the Statistical Package for the Social Sciences (SPSS) version 25.

**Results:**

A total of (49/138, 35.5%) bacteria were isolated from 138 drinking water samples, with a positive rate of (41/138, 29.7%). Among the isolates, (16/138, 11.6%) were *Staphylococcus aureus* while (33/138, 23.9%) were members of *Enterobacteriaceae*. Relatively high resistance rate among all isolates were observed for the most prescribed antibiotics in Ethiopia, including erythromycin, cotrimoxazole, doxycycline, ceftriaxone, gentamicin, and chloramphenicol. However, a low resistance was observed for early introduced antibiotics such as ciprofloxacin and recently introduced antibiotics such as cefotaxime, ceftazidime, imipenem, and meropenem. Among the 49 bacteria isolates, (32/49, 65.3%) were multidrug-resistant (MDR) pathogens while (12/49, 24.5%) were ESβL producers. Different ESβL genes were detected in most bacterial isolates. The predominant ESβL genes were bla*CTX*-M-*gp*8/25 (6/33, 18.2%), bla*CTX*-M-*gp*9 (5/33, 15.2%), and bla*CTX*-M-*gp*1 (5/33, 15.2%).

**Conclusion:**

The result of this study suggests that most water sources in the study area were contaminated by various bacterial species that are resistant to different antibiotics. Various ESβL resistance genes have also been detected. Therefore, regular sanitary inspection and bacteriological analysis should be mandatory to protect drinking water sources from contamination and the persistence of resistant bacteria.

## Introduction

High quality water is essential to people’s health, social and economic well-being. Sustainable Development Goal (SDG) 6 calls for “achieving universal and equitable access to safe and affordable drinking water for all by 2030” (1). Worldwide, 1.8 billion people still use drinking water contaminated with faeces, and this contamination occurs most frequently in Africa (2). Drinking water is often obtained from surface water, reservoirs, boreholes, and hand-dug wells (3). In developing countries such as Ethiopia, inadequate or dysfunctional sewage system coupled with sewage treatment facilities, and runoff from agricultural land and livestock production system further contribute to the contamination of such water sources (4). According to the World Health Organization, 4.2 million Ethiopians suffer from severe water scarcity (5). Majority of Ethiopians have access to only 3 to 4 liters of clean drinking water per person per day and some rural communities may travel 3 to 8 kilometers to obtain water (6). Children are particularly vulnerable to diarrheal diseases due to lack of sanitation and access to clean drinking water. According to the Ethiopian Ministry of Health, water sanitation and hygiene diseases are one of the main causes of morbidity and mortality, contributing significantly to 500,000 child deaths each year (7).

The global burden of waterborne diseases is further complicated by the increasing occurrence of antibiotic resistant genes (ARGs) observed in water environment. Antibiotic resistance genes (ARGs) that confer resistance to various antibiotics have been identified in a variety of water environments, including drinking water in developed and developing countries (8–10). The greatest risk to public health is that ARGs can be transferred from the environment to animal and human pathogens. Therefore, antibiotic-resistant bacteria (ARBs) and ARGs are considered environmental contaminant that are widely distributed in various environments, including water sources and drinking water system (11). Importantly, the rapid spread of new ARBs and ARGs around the world has accelerated in recent years due to increased release of antibiotics and other contaminants into the environment (12).

Antibiotic-resistant bacterial infection poses a serious public health threat, leading to higher illness and death rate compared to non-resistant infections. Mortality rate can increase by 50% or more with resistant strains (13). Treatment cost is significantly higher due to longer hospital stay, additional tests, and more expensive antibiotics (14), with costs per patient ranging from $18,000 to $29,000 (15). The spread of resistance through water facilitates the transmission of pathogens among human, animals, and the environment, complicating treatment and increasing healthcare cost. Therefore, the investigation of ESβL-producing *Enterobacteriaceae* and Methicillin-Resistant *Staphylococcus aureus* (MRSA) is essential due to their significant public health threats (16). Their presence in water sources can facilitate the spread of ARGs among humans, animals, and the environment, complicating treatment and increasing healthcare costs. Understanding the prevalence and transmission pathways of *Enterobacteriaceae* and MRSA in water is crucial for developing effective control measures to curb their spread and safeguard public health.

Water safety should be determined by regularly evaluating the quality of the water for the presence of organisms. The most sensitive way of determining the hygienic status of water remains routine investigation of fecal indicator organisms (5). To ensure quality water for the community, it is necessary to analyze water quality using current and up-to-date microbiological data. The frequent acute diarrhea outbreaks in some areas of the North Shoa zone (Supplementary material) indicated pathogenic bacteria might be present in drinking water sources. Information on the prevalence of potentially pathogenic bacteria and their drug resistance pattern in a rural part of North Shoa is unavailable. Therefore, this surveillance is critical to public health and safety, as it contributes and supports improvement in water quality and antimicrobial resistance control. AMR monitoring studies in Ethiopia have mainly focused on humans and animals, there is no report that showed the presence of ARBs in drinking water sources. Therefore, there is a need for research that bridges this knowledge gap. Furthermore, antimicrobial resistance (AMR) data could inform decisions and increase awareness among stakeholders and policymakers.

## Materials and method

### Description of the study area, study design and periods

A cross-sectional study design was conducted in the diarrheal hotspot area of the North Shewa zone (Minjar-Shenkora and Mojana-Wedera district) from February 01, 2022, to September 31, 2023. Minjar-Shenkora district is in the southern part of the North Shoa zone of the Amhara region. The geographical location of the study area ranged from 8°42′46″N to 9°7′37″N and from 39°12′57″E to 39°46′53″E. The topography lies at an altitude between 1,040 and 2,380 meters above sea level. There are three agro-climatic regions—Kola (24.8%), Woynadega (70.9%), and Dega (4.3%). The annual rainfall in this district is between 800–1000 mm. The Mojana-Wodera district is in the central part of the North Shoa Zone. The altitude of the study area ranges from 1,459 to 3,172 meters above sea level and is traditionally divided into three agricultural zones: Dega (28%), Woyna Dega (69%), and Kola (3%). The annual rainfall in this district is 800–1,000 mm and the annual temperature is 10–18°C. According to zonal water resource department data, hand-pump, boreholes, and wells are the primary source of drinking water in the rural North Shoa zone. The people in both districts also used surface water sources such as streams, ponds, and rivers. At the same time, the three-year zonal health department report showed water-borne diarrheal disease was the most common disease in all age groups, especially in children (Supplementary material).

### Sample collection technique and sampling frequency

A triplicate water sample was collected from 138 drinking water sources. Water sample was collected from the hand-pumps, boreholes, wells, stream water and ponds according to the WHO guideline for drinking water quality assessment (17). The sample was collected aseptically using a sterile glass bottle. During the sample collection, the inside or mouth of the bottle cap was never contacted with fingers, clothes, or unsterile items. Water (200 mL) sample from water sources was collected in 300 mL glass bottles and transported within 30 min in a cold box to Debere Berhan University, Medical Microbiology laboratory and the analysis was started immediately. The sample containing residual chlorine was neutralized by adding 0.2 mL sodium thiosulphate per 200 mL of water sample.

### Bacterial species isolation and identification

After obtaining pure colonies on MacConkey agar and recording important characteristics, *Enterobacteriaceae* were analyzed for colony morphology and pigmentation, oxidase testing, carbohydrate fermentation, H_2_S production, citrate utilization, motility, indole formation, lysine decarboxylase and lysine deaminizes production, and urea hydrolysis. *Staphylococcus aureus* was also cultured on mannitol salt agar and differentiated using colonial characteristics, catalase, coagulase, and novobiocin susceptibility testing. The interpretation of the result was carried out according to the standard protocol (18).

### Antimicrobial susceptibility testing

A standard Kirby-Bauer disk diffusion method was used to determine the antimicrobial susceptibility profile of the isolates as described by the CLSI, 2022 (19). Identified bacterial isolates were tested for their resistance pattern to antibiotics belonging to various antimicrobial classes. All antibiotics were obtained from Oxoid Ltd., UK. The antibiotics listed below were selected because they are widely prescribed in Ethiopia (Table 1).

**Table 1:** List of antibiotics used in this study.

S. no	Antibiotics and concentration	Class	Tested against
1.	Penicillin (PEN, 10 μg)	Penicillin	*S. aureus* only
2.	Ampicillin (AMP, 30 μg)		Enterobacteriaceae only
3.	Amoxicillin (AMC;30 μg)		Enterobacteriaceae only
4.	Cotrimoxazole (SXT, 30 μg)	Foliate pathway antagonist	Both
5.	Tetracycline (TET, 30 μg)	Tetracycline	
6.	Doxycycline (DXT, 30 μg)		
7.	Gentamycin (GEN, 10 μg)	Aminoglycosides	
8.	Kanamycin (KAN, 30 μg)		Enterobacteriaceae only
9.	Ciprofloxacin (CIP, 5 μg)	Fluoroquinolone	Both
10.	Chloramphenicol (CAF, 30 μg)	Phenicol	*S. aureus only*
11.	Erythromycin (ERY, 15 μg)	Macrolide	
12.	Clarithromycin (CLR, 15 μg)		
13.	Azithromycin (AZM, 30 μg)		Enterobacteriaceae only
14.	Clindamycin (CLN, 2 μg)	Lincosamides	*S. aureus*
15.	Ceftriaxone (CRO, 30 μg)	Cephalosporin	Both
16.	Cefepime (CEF, 30 μg)		Enterobacteriaceae only
17.	Cefotaxime (CTX, 30 μg)		
18.	Ceftazidime (CAZ, 30 μg).		
19.	Imipenem (IMI, 10 μg)	Carbapenem	
20.	Meropenem (MRP, 10 μg)		
21.	Aztreonam (AZT, 30 μg)	Monobactam	

### Screening of multidrug-resistant isolates

Multidrug-resistant isolates were screened according to the MDR definition, which identifies them as being resistant to one or more drugs from at least three different antimicrobial classes (20).

### Detection of β-lactamase production in *Staphylococcus aureus*

All *S. aureus* isolates were tested for β-lactamase production using the penicillin zone edge test on Mueller-Hinton agar using 10 μg penicillin disc according to CLSI guidelines (19). After incubation at 35°C for 16–18 h, sharp zone edges (cliffs) were interpreted as positive and fuzzy zones (beaches) as negative for β-lactamase production.

### Detection of methicillin resistance in *Staphylococcus aureus*

All *S. aureus* isolates were screened for methicillin resistance using cefoxitin disk (30 μg) using Kirby-Bauer disk diffusion method. Isolates with an inhibition zone of 21 mm or less around the cefoxitin disc were identified as methicillin-resistant isolates (*mecA*-mediated resistance positive), whereas isolates with an inhibition zone of more than 21 mm around the cefoxitin disc were identified as a *mecA*-mediated susceptible isolate (19).

### Confirmation of ESβL-producing bacteria

Enterobacteriaceae isolates with a ceftazidime (30 μg) inhibition zone ≤22 mm and/or a cefotaxime (30 μg) inhibition zone ≤27 mm was considered potential ESβL producers (19). To confirm ESβL production, ceftazidime (30 μg) and cefotaxime (30 μg) discs alone and in combination with clavulanic acid (30 μg/10 μg) were plated 25 mm center-to-center on Mueller-Hinton agar with bacterial suspension and incubated for (18–24 h) at 37°C. Bacterial isolates that increased the inhibition zone diameter of the combined disc by 5 mm or more compared to ceftazidime or cefotaxime disc alone were identified as ESβL producers (19).

### Detection of antimicrobial resistance genes

A molecular test was performed for detection of antimicrobial resistance gene. DNA extraction was performed to all isolates using commercial DNA extraction kit (GeneAll^®^ Ribospin^™^, South Korea) according to the manufacturer’s instructions. The quality and concentration of the extracted DNA was checked by NanoDrop spectrophotometer (Thermo Scientific). Antimicrobial resistance genes of β-lactam (*bla_CTX-M_, bla_TEM_*, *bla_SHV_*) and Carbapenemase genes (*bla_IMP_*, *bla_KPC_*, *bla_OXA-48_*, *bla*_VIM_) were determined by multiplex PCR. The amplified products were analyzed by gel electrophoresis on 2% agarose-TBE gels stained with ethidium bromide and visualized in a gel documentation system. The sets of specific primers used to detect each gene are summarized in Table 2.

**Table 2:** Primer sequences for the PCR amplification of β-lactam and carbapenemase encoding genes.

Resistance gene	5′ to 3’ primer (forward)	5′-3’ primer (reverse)	Size (bp)	Ref.
TEM	TCAGCGAAAAACACCTTG	CCCGCAGATAAATCACCA	861	20
SHV	GAGTATTCAACATTTCCGTGTC	TAATCAGTGAGGCACCTATCTC	471	20
CTX-M-1	ATGTGCAGYACCAGTAARGT	TGGGTRAARTA RGTSACCAGA	593	40
CTX-M − 9	ATGTGCAGYACCAGTAARGT	TGGGTRAARTA RGTSACCAGA	593	40
CTX-M-8/25	ATGTGCAGYACCAGTAARGT	TGGGTRAARTA RGTSACCAGA	593	40
IMP	CTACCGCAGCAGAGTCTTTG	AACCAGTTTTGCCTTACCAT	587	41
VIM	AGTGGTGAGTATCCGACAG	ATGAAAGTGCGTGGAGAC	261	41
KPC	GCTACACCTAGCTCCACCTTC	ACAGTGGTTGGTAATCCATGC	920	41
OXA-48	GCTTGATCGCCCTCGATT	GATTTGCTCCGTGGCCGAAA	281	42

Temoneira (TEM), Sulfhyldryl-variable (SHV) and Cefotaximase-München (CTX-M), Imipenemase (IMP), *Klebsiella pneumoniae* carbapenemase (KPC), oxacillin-hydrolyzing carbapenemases (OXA-types). “Y” and “R” are degenerate primer.

### Quality assurance

Before the actual work, reagents were checked for proper functioning. The sterility of the prepared culture media was checked by incubating 5% of the batch culture at 37°C overnight and observing for bacterial growth. Standard strains like *Escherichia coli* (ATCC 25922) and *Staphylococcus aureus* (ATCC 25923) were used as quality control organisms throughout the antimicrobial susceptibility study to check the performance of the disk diffusion test. For the ESβL confirmatory test, ESβL positive *K. pneumoniae* ATCC 700603 and ESβL negative *E. coli* ATCC 25922 control strains were used.

### Data processing and analysis

Data was recorded and analyzed using Statistical Package for the Social Sciences (SPSS) version 25 and frequency and percentage were used to present the data. The culture positive rate of the water sample was determined using a chi-squared (X^2^) analysis.

## Result

### Bacterial isolates

**Figure 1:**
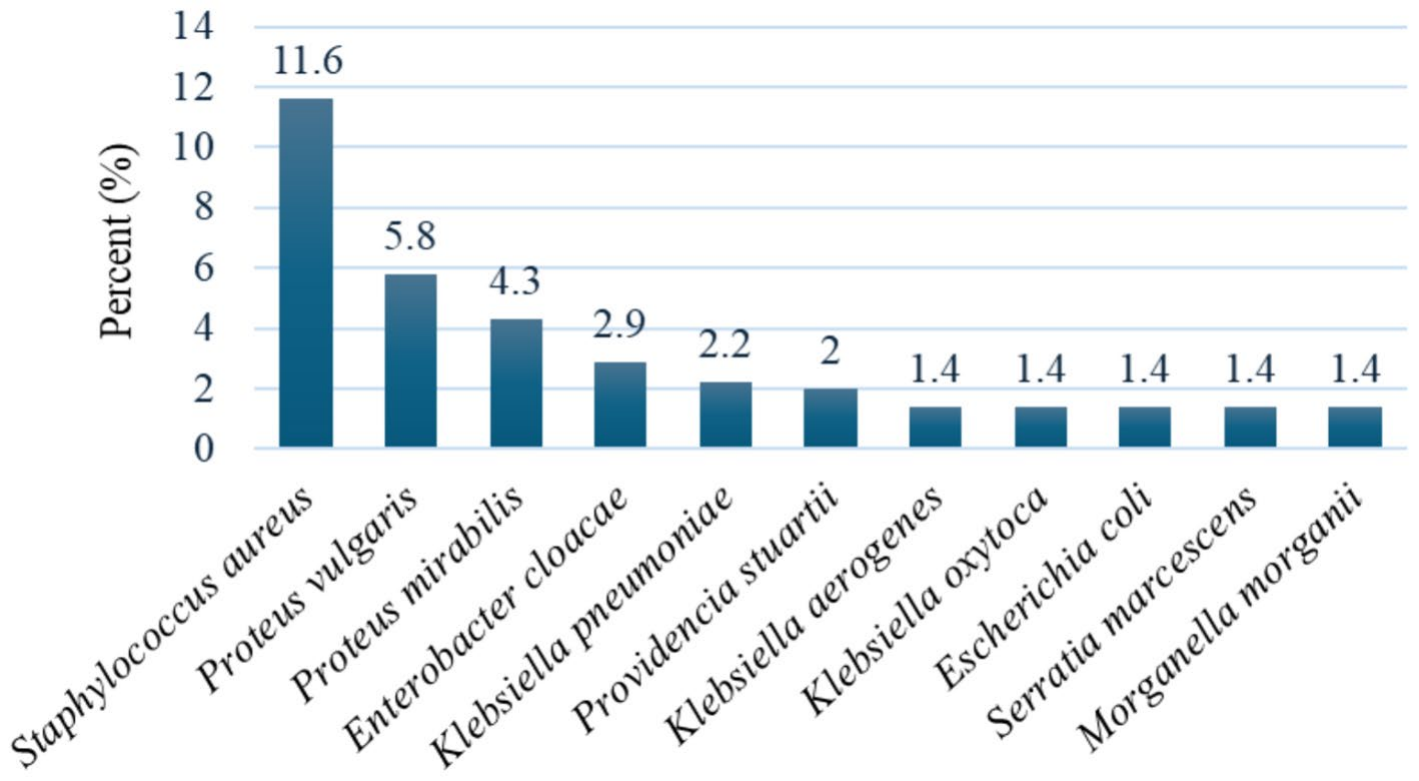
Prevalence of bacteria isolated from drinking water sources.

Out of a total of 138 drinking water samples, a total of (49/138, 35.5%) bacteria were isolated with a positive rate of (41/138, 29.7%) (OR = 1.514; 95% CI: 0.240–0.257, *p* = 0.065). The isolates were (16/138, 11.6%) *S. aureus* and (33/138, 23.9%) member of Enterobacteriaceae. Eleven different species of bacteria were isolated. The most frequent isolates were *S. aureus* (33/138, 23.9%), *Proteus vulgaris* (8/138, 5.8%) and *Proteus mirabilis* (6/138, 4.3%) (Figure 1).

### Antibiotics resistance patterns of bacterial isolates

Twenty-one different antibiotics were used in this study. Eleven different antibiotics were used for *S. aureus* and 16 different antibiotics were used for the member of *Enterobacteriaceae* isolated (Table 1). The isolated bacteria showed the highest resistance to amoxicillin, ampicillin, and cotrimoxazole. *Staphylococcus aureus* showed the highest resistance to penicillin. Relatively high resistance rates among all isolates were observed for the most prescribed antibiotics in Ethiopia, including erythromycin, cotrimoxazole, doxycycline, ceftriaxone, gentamicin, and chloramphenicol. However, low resistance was observed for early introduced antibiotics such as ciprofloxacin and recently introduced antibiotics in Ethiopia such as cefotaxime, ceftazidime, imipenem, and meropenem (Table 3).

**Table 3:** Antibiotics resistance patterns of bacterial isolates in drinking water sources.

Antibiotics, standard abbreviation and concentration	*S. aureus* (16)	*K. pneumoniae* (3)	*K. oxytoca* (2)	*E. coli* (2)	*E. cloacae* (4)	*K. aerogenes* (2)	*P. vulgaris* (8)	*P. mirabilis* (6)	*S. marcescens* (2)	*P. stuartii* (2)	*M. morganii* (2)
Penicillin (PEN, 10 μg)	12 (75)	NT	NT	NT	NT	NT	NT	NT	NT	NT	NT
Ampicillin (AMP, 30 μg)	NT	2 (66.7)	2 (100)	2 (100)	3 (75)	2 (100)	5 (62.5)	3 (50)	2 (100)	1 (50)	2 (100)
Amoxicillin (AMC;30 μg)	NT	2 (66.7)	2 (100)	2 (100)	3 (75)	2 (100)	5 (62.5)	3 (50)	2 (100)	1 (50)	2 (100)
Cotrimoxazole (SXT, 30 μg)	10 (62.5)	2 (66.7)	1 (50)	2 (100)	3 (75)	2 (100)	4 (50)	3 (50)	1 (50)	1 (50)	1 (50)
Tetracycline (TET, 30 μg)	8 (50)	1 (33.3)	1 (50)	2 (100)	3 (75)	1 (50)	3 (37.5)	3 (50)	1 (50)	1 (50)	1 (50)
Doxycycline (DXT, 30 μg)	8 (50)	2 (66.7)	1 (50)	2 (100)	3 (75)	2 (100)	4 (50)	3 (50)	1 (50)	1 (50)	1 (50)
Gentamycin (GEN, 10 μg)	6 (37.5)	1 (33.3)	2 (100)	1 (50)	2 (50)	0 (0.0)	3 (37.5)	2 (33.3)	0 (0.0)	1 (50)	1 (50)
Kanamycin (KAN, 30 μg)	NT	0 (0.0)	0 (0.0)	0 (0.0)	1 (25)	0 (0.0)	3 (37.5)	2 (33.3)	0 (0.0)	1 (50)	1 (50)
Ciprofloxacin (CIP, 5 μg)	4 (25)	0 (0.0)	0 (0.0)	0 (0.0)	1 (25)	0 (0.0)	2 (25)	2 (33.3)	0 (0.0)	1 (50)	1 (50)
Chloramphenicol (CAF,30 μg)	7 (43.8)	NT	NT	NT	NT	NT	NT	NT	NT	NT	NT
Erythromycin (ERY, 15 μg)	7 (43.8)	NT	NT	NT	NT	NT	NT	NT	NT	NT	NT
Clarithromycin (CLR, 15 μg)	6 (37.5)	NT	NT	NT	NT	NT	NT	NT	NT	NT	NT
Azithromycin (AZM, 30 μg)	NT	1 (33.3)	1 (50.0)	1 (50)	2 (50)	0 (0.0)	2 (25)	2 (33.3)	1 (50)	0 (0.0)	
Clindamycin (CLN, 2 μg)	4 (25)	NT	NT	NT	NT	NT	NT	NT	NT	NT	NT
Ceftriaxone (CRO, 30 μg)	5 (31.3)	2	2 (100)	2 (100)	2 (50)	1 (50)	3 (37.5)	2 (33.3)	1 (50)	0 (0.0)	1 (50)
Cefepime (CEF, 30 μg)	NT	2	1 (50)	1 (50)	2 (50)	0 (0.0)	0 (0.0)	0 (0.0)	1 (50)	0 (0.0)	1 (50)
Cefotaxime (CTX, 30 μg)	NT	1 (33.3)	2 (100)	1 (50)	2 (50)	0 (0.0)	0 (0.0)	0 (0.0)	0 (0.0)	0 (0.0)	1 (50)
Ceftazidime (CAZ, 30 μg).	NT	1 (33.3)	1 (50.0)	1 (50)	0 (0.0)	0 (0.0)	0 (0.0)	0 (0.0)	0 (0.0)	0 (0.0)	0 (0.0)
Imipenem (IMI, 10 μg)	NT	0 (0.0)	0 (0.0)	0 (0.0)	0 (0.0)	0 (0.0)	0 (0.0)	0 (0.0)	0 (0.0)	0 (0.0)	0 (0.0)
Meropenem (MRP, 10 μg)	NT	0 (0.0)	0 (0.0)	0 (0.0)	0 (0.0)	0 (0.0)	0 (0.0)	0 (0.0)	0 (0.0)	0 (0.0)	0 (0.0)
Aztreonam (AZT, 30 μg)	NT	0 (0.0)	1 (50.0)	0 (0.0)	1 (25)	0 (0.0)	2 (25)	2 (33.3)	1 (50)	0 (0.0)	1 (50)

NT: not tested.

### Distribution of MDR, ESβL-positive, β-lactamase production of isolates

Of the 49 bacterial isolates, (32/49, 65.3%) multidrug-resistant pathogens (MDR) and (8/49, 16.3%) were confirmed to be ESβL producer. Among the MDR isolates, *MDRs of S. aureus* (10/32, 31.25%) *and P. vulgaris* (5/32, 15.6%) were the dominant followed by *P. mirabilis* (4/32, 12.5%). Most of the isolates like *E. cloacae* (2/8, 25.0%), *K. pneumonia* (1/8, 12.5%)*, K. oxytoca* (2/8, 25.0%), *K. aerogenes* (1/8, 12.5%), and *E. coli* (1/8, 12.5%) were also among the isolates confirmed for ESβL production (Table 4). The prevalence of β-lactamase production and methicillin resistance in isolated *S. aureus* was (11/16, 68.75%) and (5/16, 31.25%) respectively.

**Table 4:** Distribution of MDR, ESβL-positive, β-lactamase production positive *S. aureus* and MRSA in drinking water sources.

Isolates	MDR	ESβL-positive	β-lactamase production positive	Methicillin resistance
*Staphylococcus aureus* (16)	10 (31.25%)	NT	11 (68.75%)	5 (31.25%)
*Klebsiella pneumoniae* (3)	2 (6.25%)	1 (12.5%)	NT	NT
*Klebsiella oxytoca* (2)	2 (6.25%)	2 (25.0%)	NT	NT
*Escherichia coli* (2)	2 (6.25%)	1 (12.5%)	NT	NT
*Enterobacter cloacae* (4)	3 (9.4%)	2 (25.0%)	NT	NT
*Klebsiella aerogenes* (2)	2 (6.25%)	1 (12.5%)	NT	NT
*Proteus vulgaris* (8)	5 (15.6%)	0	NT	NT
*Proteus mirabilis* (6)	4 (12.5%)	0	NT	NT
*Serratia marcescens* (2)	2 (6.25%)	0	NT	NT
*Providencia stuartii* (2)	1(3.13%)	0	NT	NT
*Morganella morganii* (2)	2 (6.25%)	1 (12.5%)	NT	NT
Total (49)	32 (65.3)	8 (24.5%)	–	–

NT: not tested.

### Resistance genes of ESβL-producing bacterial isolates

Overall, the predominant ESβL genes were bla*CTX*-M-*gp*8/25 (6/33, 18.2%), bla*CTX*-M-*gp*9 (5/33, 15.2%), and bla*CTX*-M-*gp*1 (5/33, 15.2%). *K. pneumoniae* strains harbour most ESβL encoding resistance gene except *VIM*, *NDM* and *SEM* while *P. vulgaris, P. mirabilis, S. marcescens, P. stuartii* and *M. morganii* did not harbour any of ESβL genes. Notably, three resistance genes were detected in isolates such as *K. oxytoca, E. cloacae and K. aerogenes*, whereas a maximum of five genes were detected in one *K. pneumoniae* isolate (Table 5).

**Table 5:** Resistance genes of ESβL-producing bacterial isolates in drinking water sources.

Resistance gene	*K. pneumoniae* (3)	*K. oxytoca* (2)	*E. coli* (2)	*E. cloacae* (4)	*K. aerogenes* (2)	*P. vulgaris* (8)	*P. mirabilis* (6)	*S. marcescens* (2)	*P. stuartii* (2)	*M. morganii* (2)	Total
TEM	2 (66.7)	/	/	/	1 (50.0)	/	/	/	/	/	3 (9.1)
SHV	1 (33.3)	1 (50.0)	/	/	1 (50.0)	/	/	/	/	/	3 (9.1)
CTX-M group-1	2 (66.7)	1 (50.0)	/	1 (25.0)	1 (50.0)	/	/	/	/	/	5 (15.2)
CTX-M group-9	2 (66.7)	1 (50.0)	/	1 (25.0)	1 (50.0)	/	/	/	/	/	5 (15.2)
CTX-M Group 8/25	2 (66.7)	1 (50.0)	1 (50.0)	1 (25.0)	1 (50.0)	/	/	/	/	/	6 (18.2)
IMP	1 (33.3)	/	/	1 (25.0)	/	/	/	/	/	/	2 (6.1)
KPC	1 (33.3)	/	/	1 (25.0)	/	/	/	/	/	/	2 (6.1)
OXA-48	1 (33.3)	/	/	1 (25.0)	/	/	/	/	/	/	2 (6.1)

Temoneira (TEM), Sulfhyldryl-variable (SHV) and Cefotaximase-München (CTX-M), Imipenemase (IMP), *Klebsiella pneumoniae* carbapenemase (KPC), oxacillin-hydrolyzing carbapenemases (OXA-types).

## Discussion

Drinking water is one of the main routes by which many infectious pathogens are transmitted to humans and cause waterborne diseases, so the quality of drinking water must be continuously monitored. The WHO recommends that water used for direct human consumption should be free of microbial contamination, as the presence of pathogens such as *E. coli* represents a potential health risk to consumers (21).

In the present study, a total of (49/138, 35.5%) bacteria were detected in a total of 138 drinking water samples, with a positive rate of (41/138, 29.7%). Like our finding, another study conducted in Ethiopia (22) found that approximately 15% of hand pump-fitted boreholes drinking water and 32 (42.6%) of swab samples from the mouth of hand pump-fitted boreholes showed positive culture result. The study conducted in South Darfur; Sudan (23) also reported drinking water contamination of 11.3%. Similarly, studies conducted in Nigeria (24) and South Africa (25) found that 100% of drinking water from hand-pump-fitted borehole sources was contaminated. Additionally, a total of 110 bacterial isolates were obtained from different drinking water sources in Ghana (10). The presence of bacteria in the drinking water sources suggests that it may be vulnerable to contamination by more harmful microorganisms and may pose a serious risk to the people who use this water.

In this study, *S. aureus* (33/138, 23.9%), *Proteus vulgaris* (8/138, 5.8%) and *Proteus mirabilis* (6/138, 4.3%) were the most frequently isolated pathogens. However, a similar study conducted in Ethiopia, showed that Klebsiella species (22) and *Escherichia coli* (26) were the predominant pathogens. Studies conducted in Sudan, Darfur (22.5%) (27) and Nigeria (33%) (9) also found *E.*

*coli* to be the main drinking water contaminant. In the studies conducted in Sudan, Darfur (27) and Nigeria (24) *E. faecalis* (20.42%) and *Klebsiella* spp. (17%) were the second and third most isolated bacteria. The same study (10) conducted in Ghana identified several bacterial species of importance to public health, including *Salmonella* spp., *Vibrio* spp., and *Klebsiella* spp. Many factors contribute to the differences in contamination rate observed across drinking water studies. Agricultural source, organic waste, irrigation water intrusion, septic tank, pit latrine around the drinking water sources, treatment barrier, and the development of biofilm within the sources are thought to be responsible for a high level of contamination faecal-borne bacteria (24, 28).

In this study, relatively high resistance rate to the most prescribed antibiotics in Ethiopia, including erythromycin, cotrimoxazole, doxycycline, ceftriaxone, gentamicin, and chloramphenicol, were observed among all isolates. Similarly, another study conducted in Ethiopia (22, 26) showed a similar pattern of resistance to these antibiotics. A study conducted in Ghana (10) also showed that all isolates showed high rates of resistance to the most prescribed antibiotics in Ghana, such as ceftriaxone and cefuroxime. The high resistance rate in Ethiopia may be due to the irrational use of antibiotics in hospitals, animal husbandry, and farms. Inappropriate use of antibiotics has been found to be very common in Ethiopian communities (29).

Of the 49 bacterial isolates, (32/49, 65.3%) were multidrug-resistant (MDR) pathogens. A similar study conducted in Ethiopia [26, 231, 35] also reported different types of MDR bacteria from drinking water sources. A study conducted in Ghana (21, 30) also reported MDR bacteria from different drinking water sources. A study conducted in Nigeria (31) also reported MDR bacterial strains isolated from water sources. Overall, the presence of MDR bacteria in drinking water sources highlights the importance of effective water quality monitoring and management practices to minimize the risk of waterborne infections and the spread of antibiotic resistance.

The prevalence of methicillin resistant *Staphylococcus aureus* (MRSA) was (5/16, 31.25%). Methicillin-resistant *Staphylococcus aureus* has been isolated from various drinking water sources in Brazil (32), Iraq (33), and Nigeria (34, 35). The presence of MRSA in drinking water sources is a serious concern due to the potential health risk it poses to humans. It is a significant pathogen associated with various infections, ranging from skin and soft tissue infections to more severe and potentially life-threatening conditions such as bloodstream infections and pneumonia. Additionally, the presence of MRSA in drinking water sources highlights the interconnectedness of environmental and human health and underscores the importance of water quality monitoring and sanitation practices.

The present study also showed that most *S. aureus isolates* (11/16, 68.75%) were positive for β-lactamase production. Similar studies conducted in Ethiopia (22, 36) and Iraq (33) showed high level of penicillin resistant *S. aureus* from drinking water sources. Beta-lactamase enzymes are produced by certain bacteria, including *S. aureus*, and they can break down β-lactam antibiotics, rendering them ineffective. Beta-lactam antibiotics include penicillin, cephalosporins, and related drugs, which are commonly used to treat bacterial infections. When *S. aureus* produces β-lactamase, it can resist the action of these antibiotics, leading to treatment failure and potentially worsening the infection.

In this study, (12/49, 24.5%) bacterial isolates were identified as ESβL producer from drinking water sources. A study conducted in Ethiopia (36) also reported that the prevalence of ESβL-producing Enterobacteriaceae was 9.4% from 64 tap water samples. A study conducted in Kinshasa, Democratic Republic of the Congo (37) also reported *Enterobacteriaceae* producing ESβL from sachet-packaged water bags. Another study conducted in Bangladesh (38) reported 17% (66/384) of the bacteria isolated from drinking water samples were ESβL producers. Identification of ESβL producers suggests that the water sources may be contaminated with fecal matter or other environmental sources harboring these resistant bacteria. This contamination can be caused by a variety of factors, including improper wastewater treatment, agricultural runoff, and improper disposal of antibiotics. Additionally, the presence of ESβL producers in drinking water has raised concerns about the effectiveness of conventional water treatment methods to remove or inactivate these bacteria. If these bacteria persist during the treatment process, they can enter the human body through consumption of drinking water and cause infections that are difficult to treat with standard antibiotics.

Different ESβL genes were detected in most bacterial isolates. The mail ESβL genes were bla*CTX*-M-*gp*8/25 (6/33, 18.2%), bla*CTX*-M-*gp*9 (5/33, 15.2%), and bla*CTX*-M-*gp*1 (5/33, 15.2%). There is no similar study conducted in Ethiopia. However, a study conducted in Kinshasa, Democratic Republic of the Congo (37) reported CTX-M1 group and SHV gene from sachet-packaged water bags. Another study conducted in Nigeria (39) also showed the presence of ESβL genes (bla*TEM*, bla*SHV* and bla*CTX*) in selected drinking water distribution channels. A similar study conducted in Ghana (10) identified different resistant genes in isolates collected from all drinking water sources. The detection of the ESβL gene in a drinking water source signals a critical need for enhanced monitoring, improved water treatment technologies, and targeted interventions to mitigate the spread of antibiotic resistance and safeguard public health. This highlights the importance of an integrated approach that considers both environmental and public health aspects in curbing antibiotic resistance.

## Conclusion and recommendations

Monitoring by traditional method (indicator organism) alone does not necessarily address the presence of human faecal contamination but constitutes one of many tools for assessing microbial water quality. This water contamination can be caused by inadequate chlorination and improper treatment. The bacterial isolates from this study indicate possible groundwater contamination. This indicates assessment of drinking water by testing total and fecal coliform alone is not sufficient. Meanwhile, the result of this study also showed the presence of a higher number of MDR, MRSA, ESβL and resistance genes in drinking water sources. Therefore, regular microbiological assessment of all drinking water sources must be carried out seasonally and health education should be given to the community for proper hygiene and waste disposal practices. The implication of MDR, MRSA, ESβL resistant bacteria, and resistant gene in a drinking water source also underscores the importance of rigorous monitoring and possibly the implementation of advanced water treatment technologies to ensure the safety of drinking water and mitigate the risk of antibiotic-resistant infections in the population.

## Data Availability

The original contributions presented in the study are included in the article/Supplementary material, further inquiries can be directed to the corresponding author.
