# Supplementary material for: Antibiotic-resistant bacteria and resistance-genes in drinking water source in north Shoa zone, Amhara region, Ethiopia
Source: Front Public Health. 2024 Sep 6;12:1422137. doi: 10.3389/fpubh.2024.1422137 (PMC11412880; doi:10.3389/fpubh.2024.1422137)
Supplement: Supplementary file 1 [file Data_Sheet_1.PDF]

Report on the number of cases over three years in North Shoa Zone, Amhara region, Ethiopia. This case is majorly reported from the two woreda/districts (Minjar-Shenkora and Mojena-wedera)

Disease with Age and Sex Distribution	July 2018 - June 2019	July 2019 - June 2020	July 2020 - June 2021	Total number of cases
Diarrhea (unspecified)	27115	32944	25535	85594
Male, < 1 year	1857	2266	1705	5828
Male, 1 - 4 years	5751	6764	5870	18385
Male, 5 - 14 years	2249	2708	1945	6902
Male, 15 - 29 years	2479	2748	1982	7209
Male, 30 - 64 years	1673	2507	1715	5895
Male, >=65 yr	516	642	381	1539
Female, < 1 year	1374	1803	1375	4552
Female, 1 - 4 years	4348	5682	4969	14999
Female, 5 - 14 years	2096	2308	1713	6117
Female, 15 - 29 years	2423	2767	1908	7098
Female, 30 - 64 years	1881	2278	1619	5778
Female, >=65 yr	468	471	353	1292
Shigellosis	10720	9874	7298	27892
Male, < 1 year	374	410	394	1178
Male, 1 - 4 years	1680	1417	1166	4263
Male, 5 - 14 years	995	843	524	2362
Male, 15 - 29 years	1468	1162	779	3409
Male, 30 - 64 years	972	1036	860	2868
Male, >=65 yr	300	245	193	738
Female, < 1 year	310	314	338	962
Female, 1 - 4 years	1323	1155	967	3445
Female, 5 - 14 years	863	783	487	2133
Female, 15 - 29 years	1188	1119	679	2986
Female, 30 - 64 years	999	1125	779	2903
Female, >=65 yr	248	265	132	645
Intestinal helminthiases	8376	10937	7183	26496
Male, < 1 year	50	49	9	108
Male, 1 - 4 years	329	286	196	811
Male, 5 - 14 years	1141	1526	953	3620
Male, 15 - 29 years	1442	1847	1098	4387
Male, 30 - 64 years	1304	1743	1092	4139
Male, >=65 yr	326	499	414	1239
Female, < 1 year	47	47	31	125
Female, 1 - 4 years	274	297	197	768

Report on the number of cases over three years in North Shoa Zone, Amhara region, Ethiopia. This case is majorly reported from the two woreda/districts (Minjar-Shenkora and Mojena-wedera)

Female, 5 - 14 years	1034	1317	894	3245
Female, 15 - 29 years	1240	1595	1105	3940
Female, 30 - 64 years	934	1320	893	3147
Female, >=65 yr	255	411	301	967
Giardiasis	4873	8127	7698	20698
Male, < 1 year	26	41	15	82
Male, 1 - 4 years	230	338	274	842
Male, 5 - 14 years	470	782	752	2004
Male, 15 - 29 years	744	1372	1235	3351
Male, 30 - 64 years	756	1384	1402	3542
Male, >=65 yr	192	289	369	850
Female, < 1 year	27	36	21	84
Female, 1 - 4 years	184	288	236	708
Female, 5 - 14 years	561	754	680	1995
Female, 15 - 29 years	873	1424	1253	3550
Female, 30 - 64 years	640	1244	1211	3095
Female, >=65 yr	170	175	250	595
Amoebiasis	3665	5428	3498	12591
Male, < 1 year	39	46	19	104
Male, 1 - 4 years	138	192	99	429
Male, 5 - 14 years	353	444	268	1065
Male, 15 - 29 years	595	812	541	1948
Male, 30 - 64 years	527	957	683	2167
Male, >=65 yr	219	303	166	688
Female, < 1 year	48	23	18	89
Female, 1 - 4 years	129	150	86	365
Female, 5 - 14 years	329	446	323	1098
Female, 15 - 29 years	678	957	513	2148
Female, 30 - 64 years	473	858	632	1963
Female, >=65 yr	137	240	150	527
Gastroenteritis (viral)	1261	2403	1387	5051
Male, < 1 year	74	172	73	319
Male, 1 - 4 years	144	354	231	729
Male, 5 - 14 years	89	178	130	397
Male, 15 - 29 years	173	248	139	560
Male, 30 - 64 years	164	249	152	565

Report on the number of cases over three years in North Shoa Zone, Amhara region, Ethiopia. This case is majorly reported from the two woreda/districts (Minjar-Shenkora and Mojena-wedera)

Male, >=65 yr	41	75	36	152
Female, < 1 year	49	115	33	197
Female, 1 - 4 years	130	271	175	576
Female, 5 - 14 years	117	185	114	416
Female, 15 - 29 years	158	265	146	569
Female, 30 - 64 years	106	220	137	463
Female, >=65 yr	16	71	21	108
Gastroenteritis (bacterial)	2547	2474	2102	7123
Male, < 1 year	156	146	69	371
Male, 1 - 4 years	454	435	346	1235
Male, 5 - 14 years	195	212	218	625
Male, 15 - 29 years	235	227	247	709
Male, 30 - 64 years	197	232	179	608
Male, >=65 yr	118	91	60	269
Female, < 1 year	170	137	73	380
Female, 1 - 4 years	399	383	267	1049
Female, 5 - 14 years	160	169	163	492
Female, 15 - 29 years	201	217	204	622
Female, 30 - 64 years	166	158	226	550
Female, >=65 yr	96	67	50	213
Typhoid Fever (due to Salmonella typhi)	5245	2494	1192	8931
Male, < 1 year	57	11	2	70
Male, 1 - 4 years	99	40	9	148
Male, 5 - 14 years	342	218	79	639
Male, 15 - 29 years	974	594	228	1796
Male, 30 - 64 years	775	408	241	1424
Male, >=65 yr	303	87	72	462
Female, < 1 year	47	8	1	56
Female, 1 - 4 years	81	21	11	113
Female, 5 - 14 years	263	156	83	502
Female, 15 - 29 years	1058	444	207	1709
Female, 30 - 64 years	958	437	212	1607
Female, >=65 yr	288	70	47	405
